# Modulation of β-Catenin Signaling by Glucagon Receptor Activation

**DOI:** 10.1371/journal.pone.0033676

**Published:** 2012-03-16

**Authors:** Jiyuan Ke, Chenghai Zhang, Kaleeckal G. Harikumar, Cassandra R. Zylstra-Diegel, Liren Wang, Laura E. Mowry, Laurence J. Miller, Bart O. Williams, H. Eric Xu

**Affiliations:** 1 Laboratory of Structural Sciences, Van Andel Research Institute, Grand Rapids, Michigan, United States of America; 2 Laboratory of Cell Signaling and Carcinogenesis, Van Andel Research Institute, Grand Rapids, Michigan, United States of America; 3 VARI/SIMM Center, Center for Structure and Function of Drug Targets, Shanghai Institute of Materia Medica, Chinese Academy of Sciences, Shanghai, People's Republic of China; 4 Department of Molecular Pharmacology and Experimental Therapeutics, Mayo Clinic, Scottsdale, Arizona, United States of America; Northwestern University Feinberg School of Medicine, United States of America

## Abstract

The glucagon receptor (GCGR) is a member of the class B G protein–coupled receptor family. Activation of GCGR by glucagon leads to increased glucose production by the liver. Thus, glucagon is a key component of glucose homeostasis by counteracting the effect of insulin. In this report, we found that in addition to activation of the classic cAMP/protein kinase A (PKA) pathway, activation of GCGR also induced β-catenin stabilization and activated β-catenin–mediated transcription. Activation of β-catenin signaling was PKA-dependent, consistent with previous reports on the parathyroid hormone receptor type 1 (PTH1R) and glucagon-like peptide 1 (GLP-1R) receptors. Since low-density-lipoprotein receptor–related protein 5 (Lrp5) is an essential co-receptor required for Wnt protein mediated β-catenin signaling, we examined the role of Lrp5 in glucagon-induced β-catenin signaling. Cotransfection with Lrp5 enhanced the glucagon-induced β-catenin stabilization and TCF promoter–mediated transcription. Inhibiting Lrp5/6 function using Dickkopf-1(DKK1) or by expression of the Lrp5 extracellular domain blocked glucagon-induced β-catenin signaling. Furthermore, we showed that Lrp5 physically interacted with GCGR by immunoprecipitation and bioluminescence resonance energy transfer assays. Together, these results reveal an unexpected crosstalk between glucagon and β-catenin signaling, and may help to explain the metabolic phenotypes of Lrp5/6 mutations.

## Introduction

G protein–coupled receptors (GPCRs) with seven-transmembrane domains form a large family that respond to extracellular signals by activating heterotrimeric G proteins. The glucagon receptor (GCGR) is a class B GPCR. Its ligand, glucagon, is a 29-amino acid peptide secreted by the islet A cells of the endocrine pancreas. The binding of glucagon to its receptor activates the cAMP/protein kinase A (PKA), protein kinase C (PKC), and mitogen-activated protein kinases (MAPK) pathways [1], [2]. The major action of glucagon is to increase glucose production from the liver by stimulating glycogenolysis and gluconeogenesis. Together with insulin, glucagon is an important regulator of glucose homeostasis.

Frizzled (Fz) receptors, which are known as atypical GPCRs, are receptors for the Wnt family of secreted glycoproteins [3]. The binding of Wnt ligands to Fz receptors activates either canonical or noncanonical Wnt pathways depending on the cellular context [4]; these are distinct from classical GPCR signaling pathways. The Wnt/β-catenin pathway is initiated by Wnt protein binding simultaneously to a Fz receptor and its co-receptor low-density-lipoprotein receptor–related protein 5/6 (Lrp5/6), causing disruption of the destruction complex which normally targets β-catenin for ubiquitin-dependent proteasomal degradation. This ultimately results in accumulation of β-catenin in the cytosol, which can then translocate into the nucleus to activate Wnt target gene expression with TCF transcription factors [5]. The canonical Wnt pathway plays an essential role in many stages of development, in stem cell renewal, and in tissue homeostasis [3], [6], [7]. Lrp5 and Lrp6 belong to a subfamily of low-density-lipoprotein receptor–related proteins that are indispensable components of the canonical Wnt signaling pathway [5].

A number of classical GPCRs have been shown to cross-talk or activate the β-catenin pathway in a Wnt-independent manner by various mechanisms. The stimulation of α-adrenergic receptors on cardiomyocytes (acting through the heterotrimeric G alpha subunit, Gq), or prostaglandin E2 receptors on colon cancer cells (acting through the G alpha subunit, Gs) results in the stabilization of β-catenin and the activation of β-catenin signaling [8], [9]. Activation of parathyroid hormone receptor type 1 (PTH1R) increases β-catenin levels and β-catenin-mediated transcription through cAMP/PKA-dependent inactivation of glycogen synthase kinase 3β (GSK-3β) in UMR106 [10] and Saos-2 mouse osteoblastic cells [11], and through both PKA and PKC dependent pathways in MC3T3-E1 cells [12]. Activation of glucagon-like peptide 1 receptor (GLP-1R) by glucagon-like peptide-1 (GLP-1) and exendin-4 (Exd4) peptides induces β-catenin signaling through the activation of cAMP/PKA and AKT pathways [13].

It was reported that binding of parathyroid hormone (PTH) to its receptor PTH1R induces activation of the β-catenin pathway through Lrp6 [14], yet another report suggested that PTH activates β-catenin signaling in a LRP5/6- and Wnt-independent manner [15]. So, whether β-catenin signaling activated through PTH1R requires Lrp6 remains a topic of debate. Mutations in Lrp5/6 are associated with bone disorders, abnormal ocular vascularization, early onset cardiovascular disease and metabolic syndrome [5], [16], [17]. These phenotypes may not all be attributable to altered responses to Wnt proteins. For instance, in eye vascularization, Lrp5 functions in a Norrin-mediated β-catenin signaling pathway [18]. Moreover, it was recently discovered that Lrp6 is not only a coreceptor for the Wnt/β-catein signaling pathway, it is also required for cAMP production for Gα_s_-coupled GPCRs including PTH1R and GCGR [19]. Because PTH1R, GLP-1R, and GCGR are well-known targets involved in regulating bone development and glucose metabolism and the genetic link between Lrp5/6 mutations and bone and metabolic disorders, we hypothesize that Lrp5/6 plays a role in mediating the β-catenin pathway induced by activation of these receptors. Consistent with previous studies on PTH1R [10], [14] and GLP-1R [13], in this study we found that activation of GCGR by glucagon also induced the β-catenin signaling pathway. Importantly, we found that Lrp5/6 is required for glucagon-induced β-catenin signaling. These results may help to explain the pleiotropic phenotypes of Lrp5 and 6 mutations and have important implications in understanding the role of Lrp5/6 in metabolic syndrome.

## Results

### Glucagon agonist induced the cAMP/PKA pathway in GCGR-expressing cells

**Figure 1:**
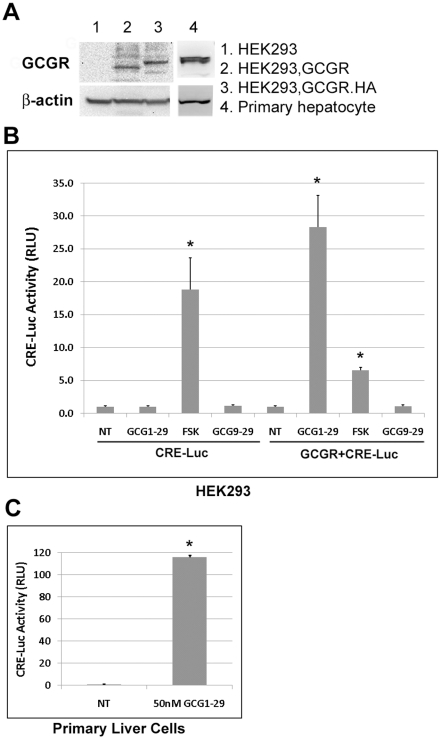
Glucagon agonist activates the CRE-Luc activity in GCGR-expressing cells. A). Protein expression for GCGR in HEK293 cells and primary hepatocytes (isolated from BL/6 mice). HEK293 cells cultured in 6-well plate were transfected with 2000 ng of pcDNA3.1 (empty vector), GCGR or HA tagged GCGR for 24 h and cells were lysed for western blot analysis as described. B). HEK293 cells cultured in 24-well plate were transfected with pcDNA3.1 and CRE-Luc (50 ng each) or CRE-Luc and GCGR plasmids (50 ng each) along with 5 ng TKRlu (an internal control) on day 1; cells were treated with or without the glucagon agonist GCG1-29 (50 nM), the antagonist GCG9-29 (50 nM) or forskolin (FSK, 10 µM) on day 2, and were harvested for luciferase activity measurements after about 17 h on day 3. Triplicate samples were used for each treatment. In all experiments, the CRE promoter–driven firefly luciferase activity was normalized to *Renilla* luciferase activity driven by the thymidine kinase promoter (for transfection control). Luc activity for non-treated group was set to 1 and Luc activities for treated groups were adjusted accordingly. *p<0.005 compared with the non-treated group. C). Primary hepatocytes cultured in 12-well plates were transfected with CRE-Luc (400 ng) and TKRlu (10 ng) plasmids on day 1, then treated with or without 50 nM GCG1-29 on day 2 and harvested on day 3 for luciferase activity measurement. Duplicate samples were used for each treatment. *p<0.001 compared with the non-treated group.

As a classical GPCR, activation of the glucagon receptor causes an increase of intracellular cAMP level, which in turn activates the PKA signaling pathway to activate cAMP-response element (CRE)-mediated gene expression. Using the CRE-Luc reporter construct, we found that HEK293 without GCGR transfection did not respond to GCG1-29 stimulation (Fig. 1B). After transfecting with GCGR, HEK293 cells became responsive to GCG1-29 (a GCGR agonist), but not to GCG9-29 (an antagonist) (Fig. 1B). As a control, forskolin, a direct PKA activator, activated CRE luciferase activity independent of GCGR expression. Using western blot, we confirmed that HEK293 cells have no detectable expression of GCGR until after transfection with a GCGR expression plasmid (Fig. 1A). These experiments suggest that HEK293 cells can be used to model GCGR signaling after ectopic expression of the receptor. We also asked if we could detect CRE luciferase activity in cells with endogenous GCGR expression. Primary liver hepatocytes are known to have endogenous GCGR expression [20]. We found that the GCG1-29 could directly activate CRE luciferase activity in primary liver cells without the need to transfect with a GCGR plasmid (Fig. 1C).

### Glucagon agonist induced β-catenin signaling in GCGR-expressing cells

**Figure 2:**
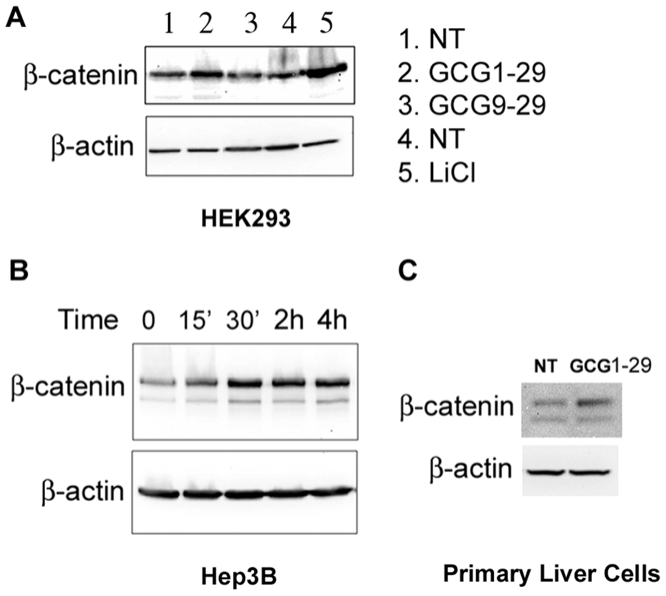
Glucagon agonist induces β-catenin stabilization in GCGR-expressing cells. A). Lanes 1–3, HEK293 cells cultured in 6-well plate were transfected with 2000 ng GCGR for 24 h and then either left non-treated(NT) or treated with 50 nM of glucagon agonist (GCG1-29) or antagonist (GCG9-29) for 1 h. Lanes 4–5, HEK293 cells cultured in 6-well plate were transfected with 2000 ng pcDNA3.1 empty vector for 24 h and then treated without or with 20 mM LiCl for 1 h. The cells were harvested and lysed, and equal amounts of protein for each sample were used for western blot analysis. The blot was first probed with anti-β-catenin antibody, then stripped and reprobed for anti-β-actin antibody as a loading control. B). Hep3B hepatocarcinoma cells were serum starved for 4 h and then treated with 50 nM GCG1-29 for the indicated time in serum-free medium. The cells were harvested and lysed. Cell lysates were used for western blot analysis. C). Mouse primary hepatocytes were treated without or with 50 nM GCG1-29 for 1 h and cells were harvested and lysed for western blot analysis.

To check whether glucagon can activate the β-catenin signaling pathway, we first used HEK293 cells transfected with the GCGR receptor. Treatment with the agonist GCG1-29 increased β-catenin protein levels relative to a control, non-treated sample (NT) or that treated with the antagonist GCG9-29 (Fig. 2A). As a positive control, treatment with lithium chloride (LiCl) also caused an increase in β-catenin levels, an indication of activation of the Wnt/β-catenin signaling pathway. To confirm this result, we also examined cells with endogenous GCGR expression, including the hepatocarcinoma cell line Hep3B and primary liver cells. Treatment of Hep3B cells with GCG1-29 caused a rapid increase of β-catenin protein levels within 15 minutes (Fig. 2B). Treatment of primary hepatocytes also caused an increase in β-catenin protein (Fig. 2C). These experiments demonstrate that activation of the GCGR receptor in cell lines and primary cells leads to β-catenin stabilization.

**Figure 3:**
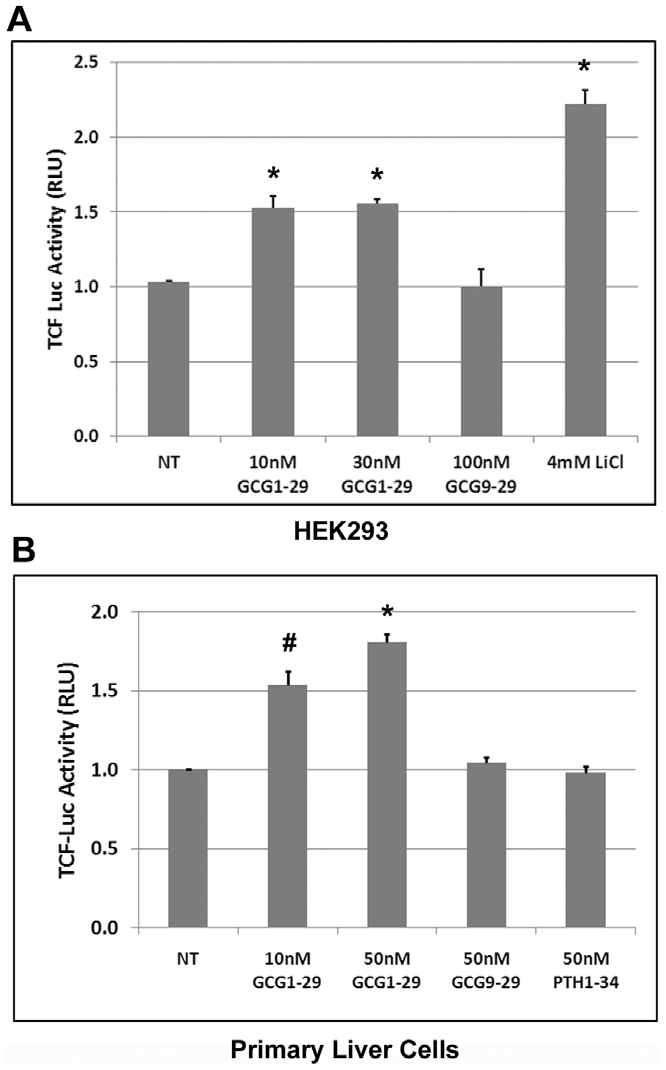
Glucagon agonist activates the β-catenin signaling in GCGR-expressing cells. A). 293STF cells cultured in 24-well plate were transfected with 100 ng GCGR and 10 ng TKRlu plasmids on day 1 and then treated with the indicated amounts of GCG1-29, GCG9-29, or LiCl, a positive control on day 2. Cells were harvested on day 3 for luciferase activity measurements. Triplicate samples were used for each group. *p<0.005 compared with the non-treated (NT) group. B). Primary liver cells cultured in 12-well plates were transfected with 400 ng TCF-Luc and 10 ng TKRlu plasmids on day 1 and then treated with the indicated amounts of GCG1-29, GCG9-29, or PTH1-34 on day 2. Cells were harvested for luciferase activity measurement on day 3. Duplicate samples were used for each treatment. ^#^p<0.02, *p<0.005 compared with the non-treated group.

Activation of the β-catenin pathway leads to stabilization of β-catenin in the cytosol, which can translocate into the nucleus and associate with TCF transcription factors to activate TCF promoter–mediated gene expression. Because we observed the stabilization of β-catenin protein upon activation of GCGR receptor, we next examined whether activation of GCGR stimulated TCF promoter–mediated luciferase activity, an indicator for an active β-catenin signaling pathway. 293STF cells (HEK293 cells stably transfected with TCF-Luc reporter DNA) [18] were transfected with the GCGR receptor and then treated with GCG1-29 or GCG9-29 peptides. We observed a small but statistically significant increase in TCF-mediated luciferase activity upon treatment with GCG1-29, but not with GCG9-29 (Fig. 3A). Treatment with LiCl also caused an increase in TCF luciferase activity (Fig. 3A). Similarly, we observed a dose-dependent increase in TCF luciferase activity in primary hepatocytes treated with GCG1-29, but not with GCG9-29 or PTH1-34 peptides (Fig. 3B). These experiments demonstrate that activation of the GCGR receptor increases TCF promoter activity. Together with the western results (Fig. 2), they demonstrated that activation of GCGR receptor leads to active β-catenin signaling.

### Coexpression of Lrp5 potentiated glucagon and GLP-1-induced β-catenin signaling

**Figure 4:**
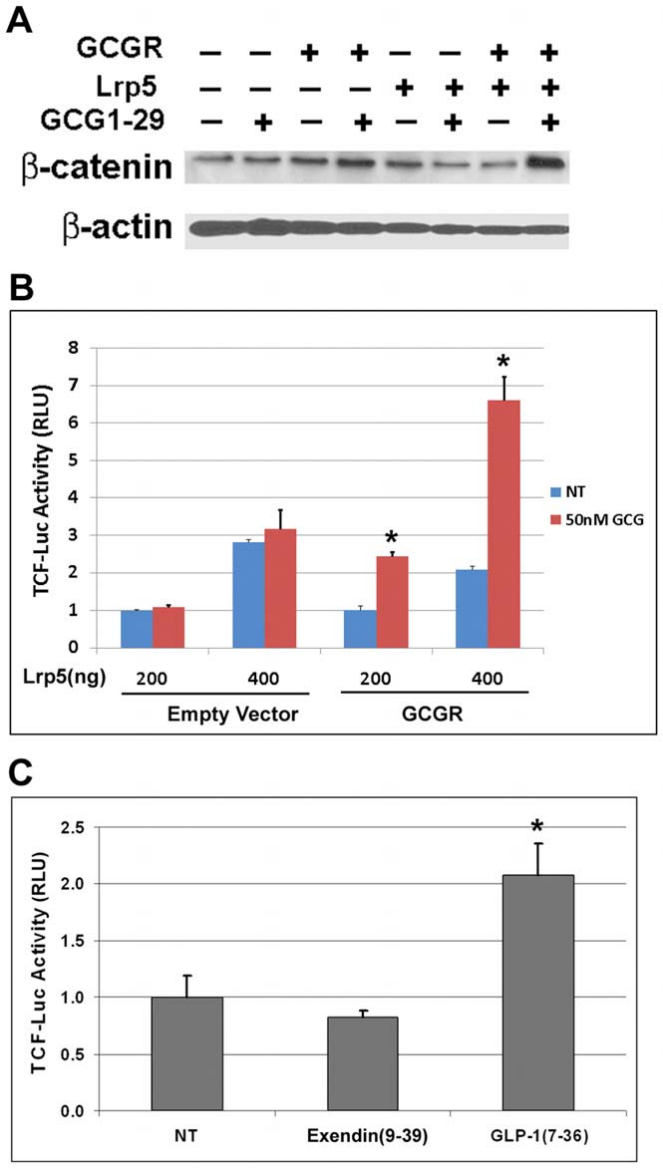
Lrp5 coexpression enhances glucagon and GLP1-induced β-catenin signaling. A). HEK293 cells cultured in 12-well plate were transfected with a combination of indicated plasmids (GCGR 1000 ng, Lrp5 500 ng) for 24 h and then treated with or without 50 nM GCG1-29 for 1 h. The cells were harvested and lysed, and samples were used for western blot analysis. The blot was first probed with anti-β-catenin antibody and then stripped and reprobed for anti-β-actin antibody as a loading control. B). 293STF cells cultured in 24-well plate were transfected with 100 ng of empty vector (pcDNA3.1) or GCGR plasmid along with the indicated amount of Lrp5 and 5 ng TKRlu plasmids on day 1, and then treated with or without 50 nM GCG1-29 on day 2. Cells were harvested for luciferase activity measurement on day 3. Triplicate samples were used for each treatment. *p<0.005 compared with non-treated group. 4C). 293STF cells cultured in 24-well plate were transfected with 100 ng GLP1R, 100 ng Lrp5 and 5 ng TKRlu plasmids on day 1 and then treated with the GLP1 agonist GLP1(7–36) (50 nM) or the antagonist Exendin(9–39) (50 nM) on day 2. Cells were harvested on day 3 to measure luciferase activity. Duplicate samples were used for each treatment. *p<0.05 compared with the non-treated (NT) group.

We observed an increase in β-catenin protein level and TCF-mediated luciferase activity upon activation of GCGR. Because Lrp5/6 is an essential coreceptor for Wnt/β-catenin signaling, we asked whether cotransfection with Lrp5/6 will potentiate glucagon-induced β-catenin signaling. HEK293 cells transfected with GCGR had a modest increase in β-catenin protein level upon GCG1-29 treatment, whereas cotransfection with GCGR and Lrp5 caused a larger increase (Fig. 4A). Next, we examined the effect of coexpression of GCGR and Lrp5 on glucagon-induced TCF luciferase activity. As expected, we observed a larger increase (2 to 3-fold vs 1.5-fold) in glucagon-induced TCF luciferase activity in HEK293 cells cotransfected with GCGR and Lrp5, relative to cells transfected with GCGR alone (Fig. 4B & 3A). As a control, transfection with high dose of Lrp5 alone also caused a small increase in TCF luciferase activity, which was not responsive to GCG1-29 treatment (Fig. 4B). Similar results were obtained when HEK293 cells were cotransfected with GCGR and Lrp6 plasmids (data not shown). Considering the report that GLP-1 also induced β-catenin signaling in GLP1R expressing cells [13], we examined the role of Lrp5 in GLP-1-induced β-catenin signaling. We found that cotransfection of HEK293 cells with GLP-1R and Lrp5 increased GLP1 agonist induced TCF luciferase activity to a similar level as observed for cotransfection of HEK293 cells with GCGR and Lrp5 (Fig. 4C). This is consistent with the hypothesis that there is a common mechanism for activation of the β-catenin signaling pathway through both GCGR and GLP1R receptors.

### Glucagon-induced TCF reporter activity was PKA-dependent

**Figure 5:**
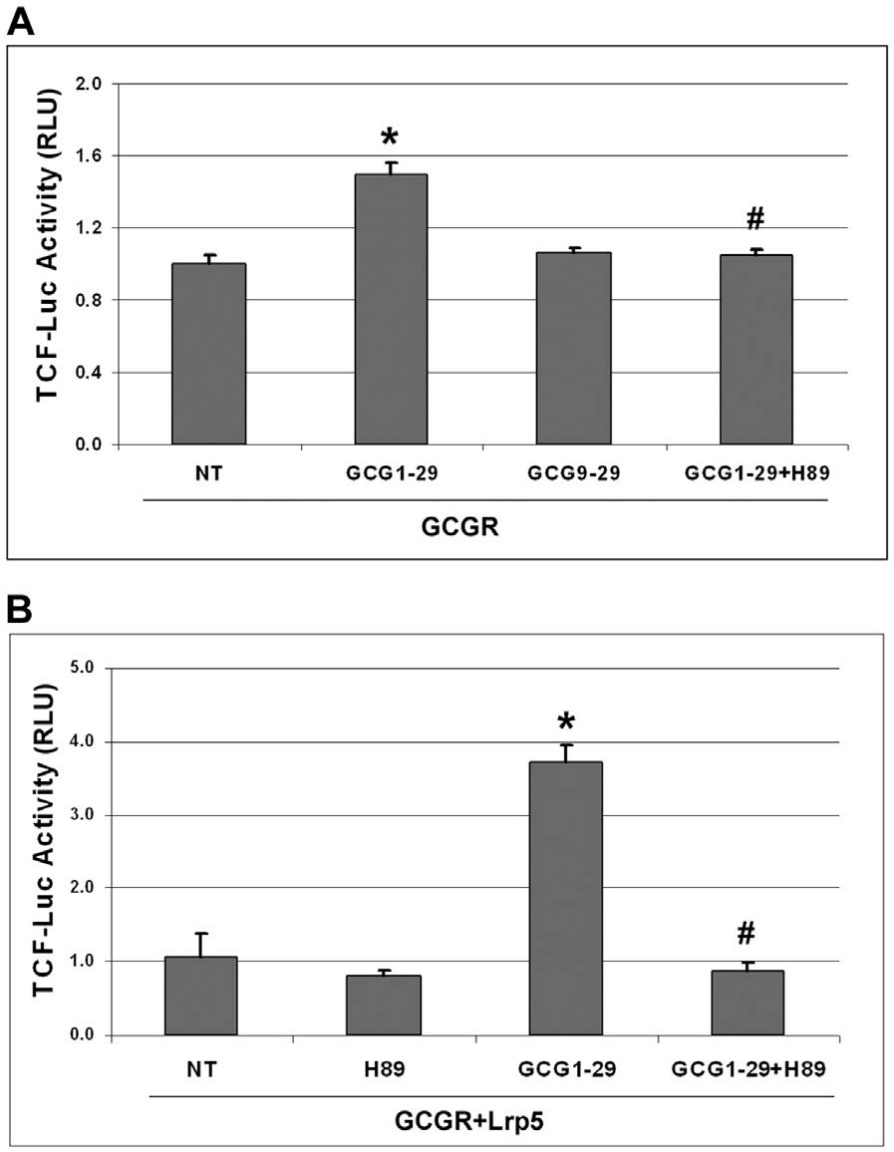
Glucagon-induced β-catenin signaling is dependent on PKA activity. A). 293STF cells cultured in 24-well plate were transfected with 100 ng GCGR and 5 ng TKRlu plasmids on day 1 and then treated with GCG1-29 (50 nM), GCG9-29 (50 nM), or GCG1-29 (50 nM) and PKA inhibitor H89 (10 µM) on day 2. Cells were harvested on day 3 to measure the TCF-mediated luciferase activity. Triplicate samples were used for each treatment. *p<0.005 compared with the non-treated group. ^#^p<0.005 compared with GCG1-29-treated group. B). 293STF cells cultured in 24-well plate were transfected with 100 ng GCGR, 100 ng Lrp5 and 5 ng TKRlu plasmids on day 1 and then treated with GCG1-29, H89, or both on day 2. Cells were harvested on day 3 to measure the TCF-mediated luciferase activity. Duplicate samples were used for each treatment. *p<0.02 compared with the non-treated (NT) group. ^#^p<0.005 compared with GCG1-29-treated group.

To understand the mechanism of glucagon-induced β-catenin signaling, we first asked whether glucagon-induced cAMP/PKA activity is required for activating the β-catenin pathway. HEK293 cells were transfected with GCGR and treated with GCG1-29 and H89, a PKA inhibitor. Inhibition of PKA activity completely blocked the activation of the β-catenin pathway induced by GCG1-29 (Fig. 5A). In another experiment, HEK293 cells were cotransfected with GCGR and Lrp5 and then treated with GCG1-29 in the presence or absence of H89 inhibitor. Treatment with H89 also completely abolished glucagon-induced β-catenin transcription activity (Fig. 5B). In these two experiments, we demonstrated that the glucagon-induced β-catenin signaling required PKA activity, consistent with previous reports on GLP-1R [13] or PTH1R [10], [11].

### Inhibition of Lrp5/6 blocked glucagon-induced TCF reporter activity

**Figure 6:**
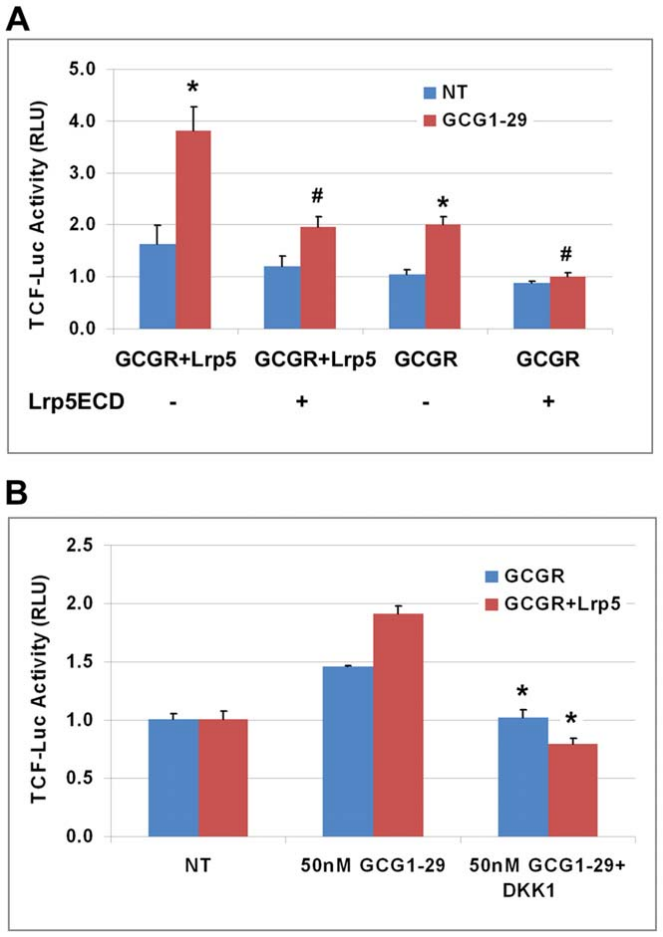
Blocking Lrp5/6 inhibited glucagon-induced β-catenin signaling. A). 293STF cells cultured in 24-well plate were transfected with a combination of GCGR (100 ng), Lrp5 (100 ng), Lrp5ECD (200 ng) and TKRlu (5 ng) plasmids as indicated on day 1, and then were treated with or without 50 nM GCG1-29 on day 2. Cells were harvested on day 3 to measure the TCF-mediated luciferase activity. Triplicate samples were used for each treatment. *p<0.005 compared with the non-treated (NT) group. ^#^p<0.005 compared with the group without the Lrp5ECD transfection. B). DKK1 protein inhibits glucagon-induced β-catenin signaling. 293STF cells cultured in 24-well plate were transfected with pcDNA3.1 and GCGR plasmids (100 ng each) or GCGR and Lrp5 plasmids (100 ng each) along with 5 ng TKRlu plasmid on day 1, and then were treated with 50 nM GCG1-29±2 µg/ml DKK1 on day 2. Cells were harvested on day 3 to measure the luciferase activity. Duplicate samples were used for each treatment. *p<0.02 compared with the GCG1-29-treated group.

We next asked whether inhibition of Lrp5/6 would reduce glucagon-induced β-catenin signaling, and we used two approaches. First, we used a dominant negative inhibitor of Lrp5/6, the Lrp5 extracellular domain (ECD) [21], [22]. Lrp5ECD inhibited glucagon-induced TCF luciferase activity when HEK293 cells were transfected with GCGR alone or GCGR+Lrp5 (Fig. 6A). In the other approach, we used Dickkopf-1 (DKK1), a known inhibitor of Lrp5/6, to block Lrp5/6 activity. In this experiment, DKK1 completely blocked glucagon-induced TCF luciferase activity when HEK293 cells were transfected with either GCGR alone or GCGR+Lrp5 (Fig. 6B). These two experiments demonstrated that inhibiting Lrp5/6 activity blocked the glucagon-induced β-catenin signaling, suggesting that Lrp5/6 is required for glucagon-induced β-catenin dependent transcription.

### Lrp5 physically interacts with GCGR

**Figure 7:**
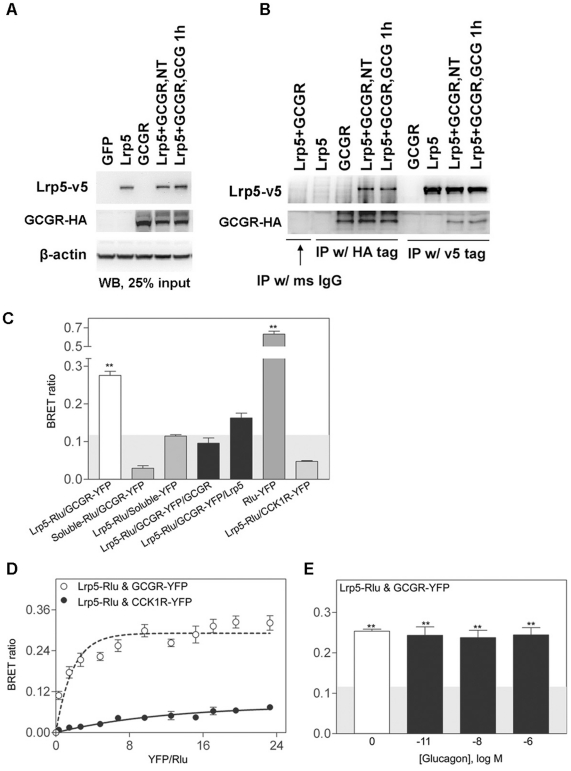
Lrp5 physically interacts with GCGR. A). HEK293 cells cultured in 6-well plate were transfected with 2000 ng of a control vector (GFP), v5-tagged Lrp5, HA-tagged GCGR, or both for 24 h and then were treated with or without 50 nM GCG1-29 for 1 h. Cells were harvested and lysed, and equal amounts of protein were used for western blot analysis. The blot was first probed with v5 antibody and then stripped and reprobed with HA antibody. The β-actin blot was used as a loading control. B). HEK293 cells were transfected and treated same as in A. The cells were harvested and lysed, and equal amounts of lysate were immunoprecipitated with the indicated antibody. For HA antibody, the antibody complex was pulled down by protein G beads. For v5 antibody, it was a single-step pull-down because the antibody was directly conjugated to the agarose beads. After pull-down, the beads were washed three times with 1× TBST and then incubated in 1× SDS sample buffer to release the bound proteins. The lysates were used for western blot analysis and probed with the indicated antibody. C–E). BRET data for Rlu-tagged Lrp5 and YFP-tagged GCGR expressed on COS-1 cells. Shown are the static BRET signals (C), saturation BRET analysis (D), and effect of natural agonist ligand binding on the BRET signals (E). Shaded area represents the background signal of 0.12 determined using Rlu-tagged Lrp5 with soluble YFP as noted. Coexpression of untagged GCGR or Lrp5 competitively reduced the BRET signals obtained between Lrp5-Rlu and GCGR-YFP (black bars). Saturation BRET analysis supported the specific interaction of Lrp5 and GCGR. The non-specific bystander type BRET signal (linear) was observed when the non-structurally-related CCK1 receptors were co-expressed with Lrp5. Incubation with the natural agonist peptide ligand, glucagon (up to 1 µM), did not significantly change the Lrp5 and GCGR BRET signal. Data are represented as the means ± S.E.M. from four to six independent experiments performed in triplicate. Data marked with ** were significantly different from background signals at the level of p<0.01.

Because cotransfection with GCGR and Lrp5 increases glucagon-induced β-catenin stabilization and TCF luciferase activity, we examined whether GCGR and Lrp5 physically interact with each other by immunoprecipitation. HEK293 cells were cotransfected with HA-tagged GCGR and v5-tagged Lrp5. Using western blots, we found that both proteins were well expressed (Fig. 7A). If we immunoprecipitated a cell lysate using anti-HA antibody to pull down HA-tagged GCGR, Lrp5 was co-immunoprecipitated (Fig. 7B). Consistent with this, using v5 antibody to pull down v5-tagged Lrp5, we also pulled down GCGR protein. We also saw a diffused band above the band with expected molecular weight for GCGR in the immunoprecipitated samples (Fig. S5), which may be a nonspecific band picked up by the HA antibody. We found that the association of GCGR and Lrp5 was independent of GCG1-29 treatment. As controls, immunoprecipitation with normal mouse IgG did not pull down either protein; further, anti-HA antibody did not pull down v5-tagged Lrp5 and anti-v5 antibody did not pull down HA-tagged GCGR. Similarly, GCGR was co-immunoprecipitated with Lrp6 in a glucagon-independent manner (Fig. S1). To further confirm the immunoprecipitation results, we used bioluminescence resonance energy transfer (BRET) assay to examine GCGR and Lrp5 interaction by tagging GCGR with YFP and Lrp5 with Rlu, respectively. In the static BRET assay, we found that GCGR interacts with Lrp5 with a BRET ratio of 0.28, which is significantly above the background of 0.12 (Fig. 7C). As a negative control, Lrp5 did not interact with the non-structurally-related CCK1 receptor. Also coexpression of untagged GCGR or Lrp5 competitively inhibited the BRET signal between Rlu-tagged Lrp5 and YFP-tagged GCGR (Fig. 7C). The positive BRET signal was further confirmed by saturation BRET studies that showed an increase in BRET signal reaching a plateau, which is indicative of the existence of an oligomeric complex containing GCGR and Lrp5 (Fig. 7D). As a negative control, a linear curve was observed for the CCK1R and Lrp5 BRET signal. Lastly, we checked the effects of occupation of the GCGR with its natural agonist ligand, glucagon, on the interaction between GCGR and Lrp5 (Fig. 7E). We did not find any significant difference in the intensity of the BRET signal with glucagon concentrations up to 1 µM, which is well above the saturated concentrations required to elicit full response. In summary, the immunoprecipitation data and BRET assay data supports the interpretation that GCGR interacts with Lrp5 in a ligand-independent manner and ligand treatment did not significantly change their interaction.

## Discussion

In this report, we found that activation of the glucagon receptor not only led to activation of the classic cAMP/PKA pathway (Fig. 1), but also activated β-catenin signaling (Fig. 2 and 3). This activity was specific for glucagon agonists as glucagon antagonists or other peptides did not elicit the same response (Fig. 2 and 3). Activation of the β-catenin pathway by glucagon was very rapid (within 30 min, Fig. 2B), suggesting it is likely to be a direct effect. This is consistent with other reports which demonstrate that activation of several GPCRs, including PTH1R [10], [11], [12] and GLP-1R [13], leads to up-regulation of the β-catenin pathway [8], [9], [23].

PTH1R, GLP-1R and GCGR all belong to class B GPCR family, which have the closest phylogenetic relationship to Frizzled receptors [24]. Similar to the Frizzled receptors, these GPCRs, as reported previously and here, may partner with Lrp5/6 to mediate β-catenin activation. Lrp6 was reported to play a direct role in mediating stabilization of β-catenin after exposure of cells to PTH [14]. We found that Lrp5/6 also has an important role in glucagon-induced β-catenin signaling. First, cotransfection of GCGR and Lrp5/6 increased glucagon-induced β-catenin stabilization (Fig. 4A) and β-catenin-mediated transcriptional activity (Fig. 4B). Second, inhibiting Lrp5/6 function by a dominant negative construct (Lrp5ECD) or a functional inhibitor (DKK1) blocked glucagon-mediated β-catenin signaling (Fig. 6). One study showed that PTH1R activated β-catenin signaling is independent of Lrp5/6 [15]. However, this conclusion is based on lower expression levels of Lrp5/6 in Chinese Hamster Ovary (CHO) cells and could reflect cell-type specific differences. An involvement of Lrp6 for PTH [14] and of Lrp5/6 for glucagon mediated β-catenin signaling may indicate that there is a common mechanism of signaling for some class B GPCRs including PTH1R, GLP-1R, and GCGR. Indeed, cotransfection of Lrp5 can also enhance GLP-1 peptide–mediated cross-talk to β-catenin signaling (Fig. 4C).

Using immunoprecipitation, we found that Lrp5/6 physically interacted with GCGR (Fig. 7A–B, S1). Using BRET assay, we further confirmed that ectopic expressed GCGR and Lrp5 do interact specifically on the cell surface (Fig. 7 C–E). We found that this interaction is ligand-independent in both experiments, which is somewhat different from previous report which showed that PTH1R interacts with Lrp5 in a ligand-independent manner, but with Lrp6 in a ligand-dependent manner [14]. This difference may be due to different receptors, cellular contexts or experimental conditions. Considering that PTH1R and GCGR are different receptors that can interact with Lrp5/6, one model is that these interactions may occur via a common adaptor protein to which GCGR, PTH1R, and Lrp5/6 all can bind, e.g. a G-protein complex.

Our data suggested that association of GCGR and Lrp5 alone is not sufficient for activation of the downstream β-catenin pathway. In addition, ligand binding is required, presumably through inducing conformational changes of GCGR and phosphorylation of Lrp5/6 to activate the downstream β-catenin pathway. However, pre-association of GCGR with Lrp5/6 on the cell surface can greatly facilitate the signaling communications between GCGR and Lrp5/6. So activation of GCGR upon ligand binding can directly cross-talk to Lrp5/6 to transmit downstream β-catenin signaling whereas phosphorylation and activation of Lrp5/6 on the other hand can communicate back to GCGR to boost GCGR mediated cAMP/PKA pathway. This mutual communication is supported by our cell-based reporter data showing that cotransfection of Lrp5 not only enhanced glucagon induced β-catenin signaling (Fig. 4) but also enhanced glucagon induced cAMP/PKA signaling (Fig. S2). It is also consistent with recent studies with PTH1R showing that Lrp6 is not only required for PTH mediated β-catenin signaling pathway [14], but also promotes cAMP/PKA signaling [19]. We found that glucagon-induced β-catenin signaling was dependent on PKA activity (Fig. 5), which is consistent with other reports for class B GPCRs such as PTH1R [10], [11], [12], [14] and GLP-1R [13] and suggests that the β-catenin pathway and cAMP/PKA pathway are interconnected. This is different from Wnt protein–induced β-catenin pathway, which does not require PKA activity [14]. Interestingly, treatment of GCGR and Lrp5 expressing cells with glucagon and Wnt3a conditioned media had a synergistic effect on the β-catenin signaling pathway, suggesting that the cAMP/PKA pathway and the β-catenin pathway reinforce each other (Fig. S4).

Glucagon-induced β-catenin signaling is relatively weaker than Wnt protein-induced β-catenin signaling. The relative weak signal was not due to lack of interaction between GCGR and Lrp5/6, but may be intrinsic to GCGR itself. In Frizzled receptors, two residues in the intracellular loops 1 and 3 (Arg263 and Leu443 in human Fz5) and a motif in the C-terminal tail (KTXXXW) play an important role in Dishevelled protein recruitment and Wnt/β-catenin signaling [25], [26]. Sequence analysis indicated that GCGR lacks these key residues of Frizzled receptors in its intracellular loops (Fig. S3). The C-terminal motif is not completely conserved in GCGR (RRXXXW in GCGR, Fig. S3). Note that for PTH1R, this motif is better conserved (KSXXXW), which may allow better binding to Dishevelled and more robust β-catenin signaling for PTH1R.

What is the physiological consequence of cross-talk to β-catenin signaling from GCGR? Wnt/β-catenin signaling helps to promote stem cell renewal and in many cases favors proliferation over differentiation. Several lines of evidence suggest that Wnt/β-catenin signaling may help pancreatic cells survive and proliferate. First, Wnt/β-catenin signaling is involved in the genesis of pancreatic islets and the proliferation of pancreatic beta cells [27]. Second, polymorphisms in the TCF7L2 gene, one of the LEF/TCF family members that bind and mediate β-catenin activity in the nucleus, are highly associated with the risk of type 2 diabetes [28]. Thus activation of the β-catenin signaling pathway by GLP1 peptide may contribute to regulation of pancreatic islet cell proliferation [13]. Given the similarity between GLP1R and GCGR, we speculate that glucagon-mediated activation of β-catenin signaling may play a similar role in regulating liver cell proliferation and regeneration. During development, Wnt/β-catenin signaling often occurs in a temporal and restricted manner, and overactivation of this pathway leads to cancer development. Thus, the relative weak signal may be beneficial to prevent cancer development, while still allowing for self-renewal of specific cellular populations.

Overall, our results and others suggested that the function for Lrp5/6 is broader than being a coreceptor for the canonical Wnt signaling pathway and it can serve as a key scaffold molecule to participate in both β-catenin and cAMP/PKA signaling pathways. By engaging with several different GPCRs, it can regulate a range of important physiological functions including embryonic development, bone formation, and metabolism. Our results may help to explain the pleiotropic phenotypes of Lrp5 and 6 mutations. To better understand the physiological role of Lrp5/6 in metabolism, future studies with Lrp5/6 conditional knockout or mutant mice are needed.

## Materials and Methods

### Ethics statement

All animal work has been carried out in accordance with US National Institutes of Health guidelines. All procedures utilized in this study were evaluating for animal welfare and ethics considerations and were approved by the VARI Institutional Animal Care and Use Committee (VARI-IACUC) prior to initiation (VAI-IACUC protocol ID #09-10-025).

### Reagents

LiCl was purchased from Sigma Aldrich. The antibodies used were mouse anti-β-catenin monoclonal antibody (BD Biosciences), mouse anti-β-actin antibody (Abcam), mouse anti-v5 antibody and v5 antibody-conjugated agarose beads (Sigma Aldrich), rabbit anti-GCGR antibody (H-57) (Santa Cruz), and mouse anti-HA antibody (Covance). Forskolin and H89 were obtained from Calbiochem. A Dual Luciferase assay kit was purchased from Promega. GLP-1 peptides, glucagon peptides, and PTH peptides were purchased from peptide 2.0. Dickkopf-1 (DKK1) protein was purchased from R&D systems.

### DNA plasmids

The TKRlu plasmid with the *Renilla* luciferase (Rlu) gene under the control of the thymidine kinase promoter was used as a transfection control (Promega). The TCF-Luc plasmid (M50 Super 8× TOPFlash), with the firefly luciferase gene under the control of the TCF promoter, was obtained from Addgene. Mouse DKK1, Lrp5, Lrp6, and Wnt5-Fz8 plasmids have been described before [29], [30]. The CRE-Luc plasmid (pGL4.29[luc2p/CRE/Hygro]), with the firefly luciferase gene under the control of the CRE promoter, was obtained from Promega. The GCGR plasmid was obtained from Missouri S&T cDNA Resource Center. For immunoprecipitation, GCGR DNA was cloned into the pcDNA3.1 expression vector with three tandem HA tags at the C-terminus by PCR. The Lrp5 extracellular domain (ECD, residue 32-1375) was cloned into pcDNA6 vector with an Ig κ leader sequence at the N-terminus to allow its secretion into media supernatant. For BRET studies, the yellow fluorescent protein (YFP) gene was cloned into the C-terminus of GCGR to create the GCGR-YFP fusion construct. The Rlu gene was cloned into the C-terminus of Lrp5 to create Lrp5-Rlu fusion construct. All constructs were verified by automated DNA sequencing.

### Cell lines, mice, and primary cell preparation

HEK293, COS-1 and Hep3B cells were from ATCC. 293STF cells (which are HEK293 cells with an integrated “Super-Top-Flash” TCF-luciferase reporter) have been described [18]. C57Bl/6J mice were purchased from The Jackson Laboratory (Bar Harbor, ME). Mice were housed under specific pathogen-free conditions in micro-isolator cages under an American Association for Laboratory Animal Accreditation and Certification–approved protocol. Four- to six-week-old mice were used to isolate the primary hepatocytes. The procedures for isolating primary hepatocytes have been described previously [31].

### Western blot analysis

HEK293 or other cells were cultured in 6-well plates (Costar, Cambridge, MA). For experiments requiring transfection, cells were transfected with the indicated DNA using Lipofectamine 2000 (Invitrogen) according to the manufacturer's protocol. After transfection, cells were treated with different reagents for the indicated time. Cell pellets were lysed in 90 µl of 1× cell lysis buffer (Cell Signaling Technology) on ice for 30 min and western blots were performed as described earlier [32]. The blots were developed with Pico Chemiluminescence substrate (Pierce Biotechnology) and exposed to Kodak X-O mat films, which were scanned on an HP ScanJet flat-bed scanner (Hewlett Packard). Alternatively, the blots were exposed by using a Biorad Quantity One Gel Box (Biorad). For reprobing, membranes were stripped using a solution containing 62.5 mM Tris-HCl, 2% SDS, and 100 mM β-mercaptoethanol at 62°C for 45 min.

### Immunoprecipitation

HEK293 cells in 6-well plate were transfected with HA-tagged GCGR with or without v5-tagged Lrp5 plasmids on day 1 with Lipofectamine 2000 (Invitrogen) according to the manufacturer's protocol. On day 2, cells were treated with or without GCG1-29 for 1 h. Cells were subsequently harvested and lysed in 100 µl of 1× cell lysis buffer (Cell Signaling Technology) on ice for 30 min. Cell debris was removed by centrifugation. The ExactaCruz™ E kit from Santa Cruz was used to immunoprecipitate HA-tagged GCGR from the cell lysate with 2–5 µg mouse anti-HA antibody according to the manufacturer's protocol. For immunoprecipitation with v5 antibody, 50 µl of anti-v5 agarose beads were directly incubated with the cell lysate for 2 h at 4°C with mixing induced by rotation of the tube. The beads were spun down and washed three times with 1× TBST (Tris-buffered saline with 0.05% Tween 20) buffer. Then 50–60 µl of 2× reducing electrophoresis buffer was added to the beads and they were incubated at 37°C for 5 min before loading onto an SDS-gel for western blot analysis.

### Luciferase assay

To measure the CRE reporter activity, HEK293 cells were transfected with CRE-Luc DNA. To measure the TCF reporter activity, either 293STF cells or HEK293 cells transfected with the TCF-Luc plasmid were used. HEK293 or 293STF cells were maintained in Dulbecco's modified Eagle's medium (DMEM) with 5% fetal bovine serum (FBS). Cells were plated on a 24-well plate for 24 h prior to transfection. Cells were transfected in Opti-MEM I (Invitrogen) with various plasmids+10 ng of control TKRlu plasmid (constitutive expression of *Renilla* luciferase) by use of Lipofectamine 2000 (Invitrogen) according to the manufacturer's protocol. Cells were induced with the indicated reagents at 24 h after transfection. Seventeen hours after induction, cells were harvested and lysed in the plate with 100 µl of 1× passive lysis buffer (Promega) at room temperature for 15 min, and the firefly and *Renilla* luciferase activities were measured on an Envision luminometer (Perkin-Elmer) with the Dual Luciferase assay kit (Promega). Firefly luciferase data were normalized to *Renilla* luciferase data.

### BRET studies

BRET experiments were performed using COS-1 cells as described previously [33]. Cells were seeded at a density of 0.5×10^6^ cells/dish in sterile 10-cm tissue culture dishes in DMEM supplemented with 5% Fetal Clone II (Logan, UT). After 24 h, the cells were transiently transfected with 3 µg of DNA/dish using the diethylaminoethyl (DEAE)-dextran method [34]. Cells were used 48–72 h later. Receptor-bearing COS-1 cell suspensions of approximately 25,000 cells/well were used for bioluminescence and fluorescence measurements in 96-well Optiplates. BRET assays were initiated by mixing 5 µM coelenterazine *h* (*Renilla* luciferase-specific substrate) (Biotium, Hayward, CA) with the cell suspension. The luminescence signals were collected immediately using a 2103 Envision fluorescence plate reader configured with the <700 nm dichroic mirror and with dual emission filter sets for luminescence (460 nm, bandwidth 25 nm) and fluorescence (535 nm, bandwidth 25 nm). Fluorescence of the YFP was acquired by exciting the samples at 485 nm and collecting the emission at 525 nm. The BRET ratios were calculated based on the ratio of emission from YFP and Rlu, as described previously [33].

Saturation BRET studies were also performed as described previously [33]. In brief, COS-1 cells were transfected with a fixed concentration of Rlu-tagged constructs as donor (1.5 µg DNA/dish) and with increasing concentrations of YFP-tagged constructs as acceptors (0.3 µg to 6 µg DNA/dish). After 48–72 h, BRET assays were performed. The BRET signals were plotted as ratios relative to the ratios of emissions of YFP/Rlu, and the curve fit was evaluated based on R^2^ values using Prism 4.0. (GraphPad, San Diego, CA).

## Supporting Information

Figure S1 **Lrp6 physically interacts with GCGR.** A). HEK293 cells were transfected with a control vector (GFP), v5-tagged Lrp6, HA-tagged GCGR, or both for 24 h and then were treated with or without 50 nM GCG1-29 for 1 h. Cells were harvested and lysed and used for western blot analysis. The blot was probed with v5 antibody and then stripped and reprobed with HA antibody. The β-actin blot was used as a loading control. B). HEK293 cells were transfected and treated similarly as in A. The cells were harvested and lysed, and equal amounts of lysate were immunoprecipitated with the indicated antibody. For HA antibody, the antibody complex was pulled down by protein G beads. For v5 antibody, it was a single-step pull-down because the antibody was directly conjugated to the agarose beads. After pull-down, the beads were washed three times with 1× TBST and then incubated in 1× SDS sample buffer to release the bound proteins. The lysates were used for western blot analysis and probed with the indicated antibody.
(TIF)

Figure S2 **Coexpression of Lrp5 enhanced the CRE Luciferase activity.** HEK293 cells were transfected with GCGR or GCGR+Lrp5 plasmids along with CRE-Luc and TKRlu (an internal control) on day 1. Cells were left untreated or treated with 50 nM GCG1-29 on day 2. Cells were harvested on day 3 to measure the luciferase activity as described.
(TIF)

Figure S3 **Sequence alignment of the intracellular loop 1 and 3, C-terminal region of Frizzled receptors and three class B GPCRs.** The IC loops and C-terminal region were predicted by the HMMTOP server [35] and aligned by clustalW program [36]. The conserved residues critical for activation of Wnt/β-catenin signaling are highlighted in yellow based on previous studies [25]. Single mutations abolish Wnt/β-catenin signaling activity of human Frizzled 5 (Fz5) are indicated on the top of the alignment [25]. Residue number corresponds to human Fz5 sequence.
(TIF)

Figure S4 **Effects of glucagon and Wnt3a on the TCF Luc reporter activity in the 293STF cells expressing GCGR and Lrp5.** 293STF cells were transfected with 100 ng each of Lrp5 and GCGR plasmids and 10 ng of TKRlu (an internal control) on day 1. Cells were either left untreated, or treated with 50 nM GCG1-29, 10% Wnt3a Conditioned Media (CM) or 50 nM GCG1-29+10% Wnt3a CM on day 2. Cells were harvested on day 3 to measure the luciferase activity as described in the “materials and methods” section. The data label indicated the fold of induction compared with the non-treated group (NT).
(TIF)

Figure S5 **The immunoprecipitation experiment using HA antibody.** HEK293 cells were transfected with 2000 ng of the indicated DNAs and treated with or without 50 nM GCG1-29 for the indicated time, similarly as in Fig. 7. Cells were harvested and lysed, and cell lysates with equal amounts of protein were used for western blot analysis for Lane 1–3. For lane 4–10, the cells were harvested and lysed, and equal amounts of lysate were immunoprecipitated with HA antibody. The antibody complex was pulled down by protein G beads. After pull-down, the beads were washed three times with 1× TBST and then incubated in 1× SDS sample buffer to release the bound proteins. The lysates were used for western blot analysis.
(TIF)
