# Supplementary figures and images for: Modulation of β-Catenin Signaling by Glucagon Receptor Activation
Source: PLoS One. 2012 Mar 16;7(3):e33676. doi: 10.1371/journal.pone.0033676 (PMC3306284; doi:10.1371/journal.pone.0033676)

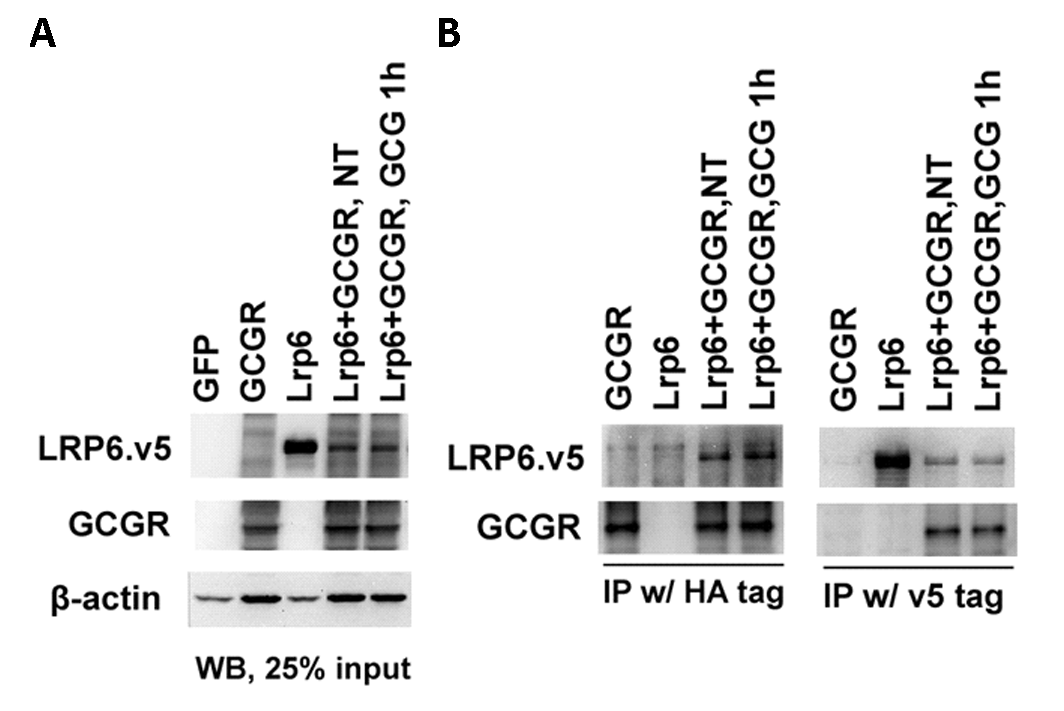

Supplement: Figure S1 — Lrp6 physically interacts with GCGR. A). HEK293 cells were transfected with a control vector (GFP), v5-tagged Lrp6, HA-tagged GCGR, or both for 24 h and then were treated with or without 50 nM GCG1-29 for 1 h. Cells were harvested and lysed and used for western blot analysis. The blot was probed with v5 antibody and then stripped and reprobed with HA antibody. The β-actin blot was used as a loading control. B). HEK293 cells were transfected and treated similarly as in A. The cells were harvested and lysed, and equal amounts of lysate were immunoprecipitated with the indicated antibody. For HA antibody, the antibody complex was pulled down by protein G beads. For v5 antibody, it was a single-step pull-down because the antibody was directly conjugated to the agarose beads. After pull-down, the beads were washed three times with 1× TBST and then incubated in 1× SDS sample buffer to release the bound proteins. The lysates were used for western blot analysis and probed with the indicated antibody. (TIF) [file pone.0033676.s001.tif]

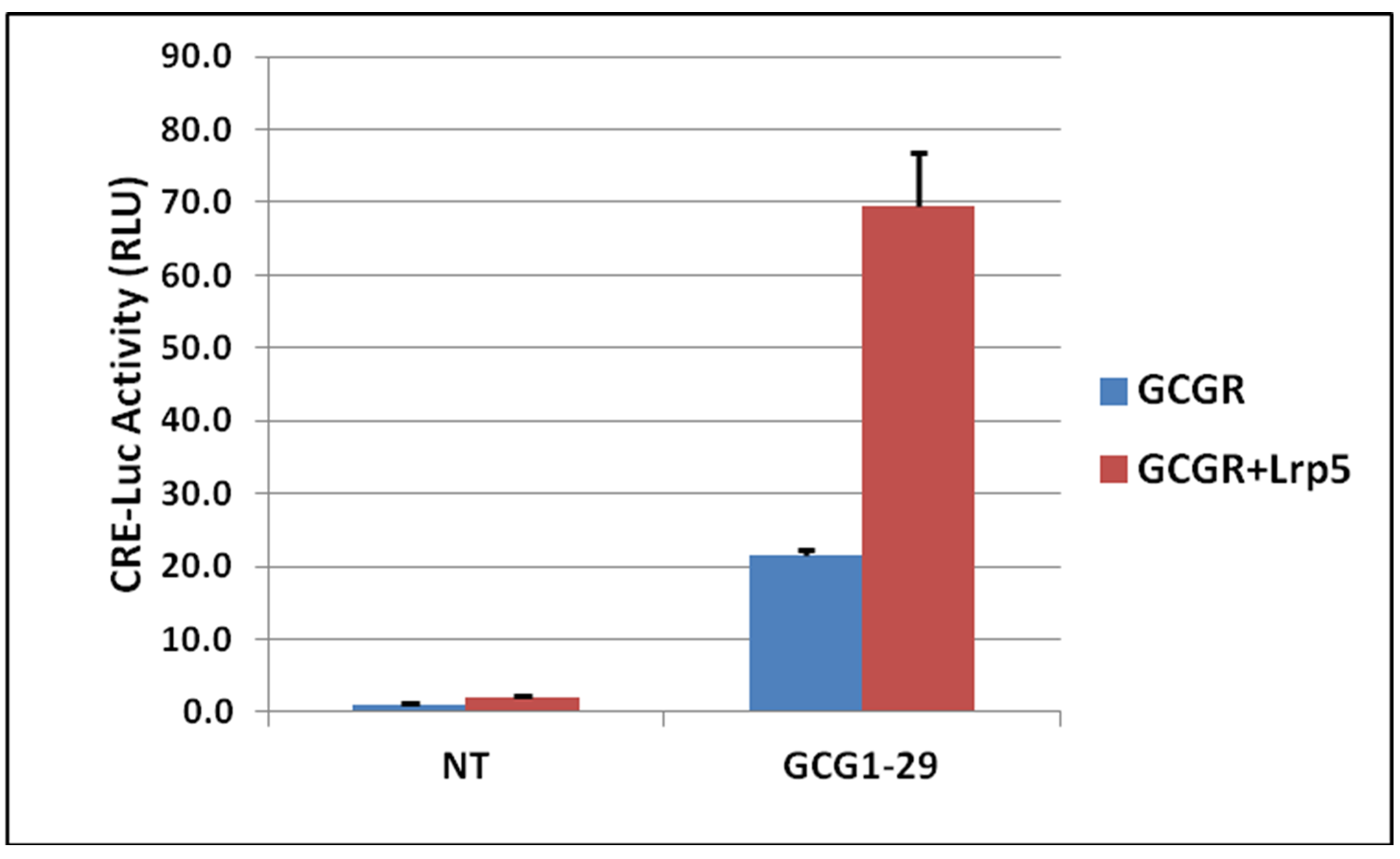

Supplement: Figure S2 — Coexpression of Lrp5 enhanced the CRE Luciferase activity. HEK293 cells were transfected with GCGR or GCGR+Lrp5 plasmids along with CRE-Luc and TKRlu (an internal control) on day 1. Cells were left untreated or treated with 50 nM GCG1-29 on day 2. Cells were harvested on day 3 to measure the luciferase activity as described. (TIF) [file pone.0033676.s002.tif]

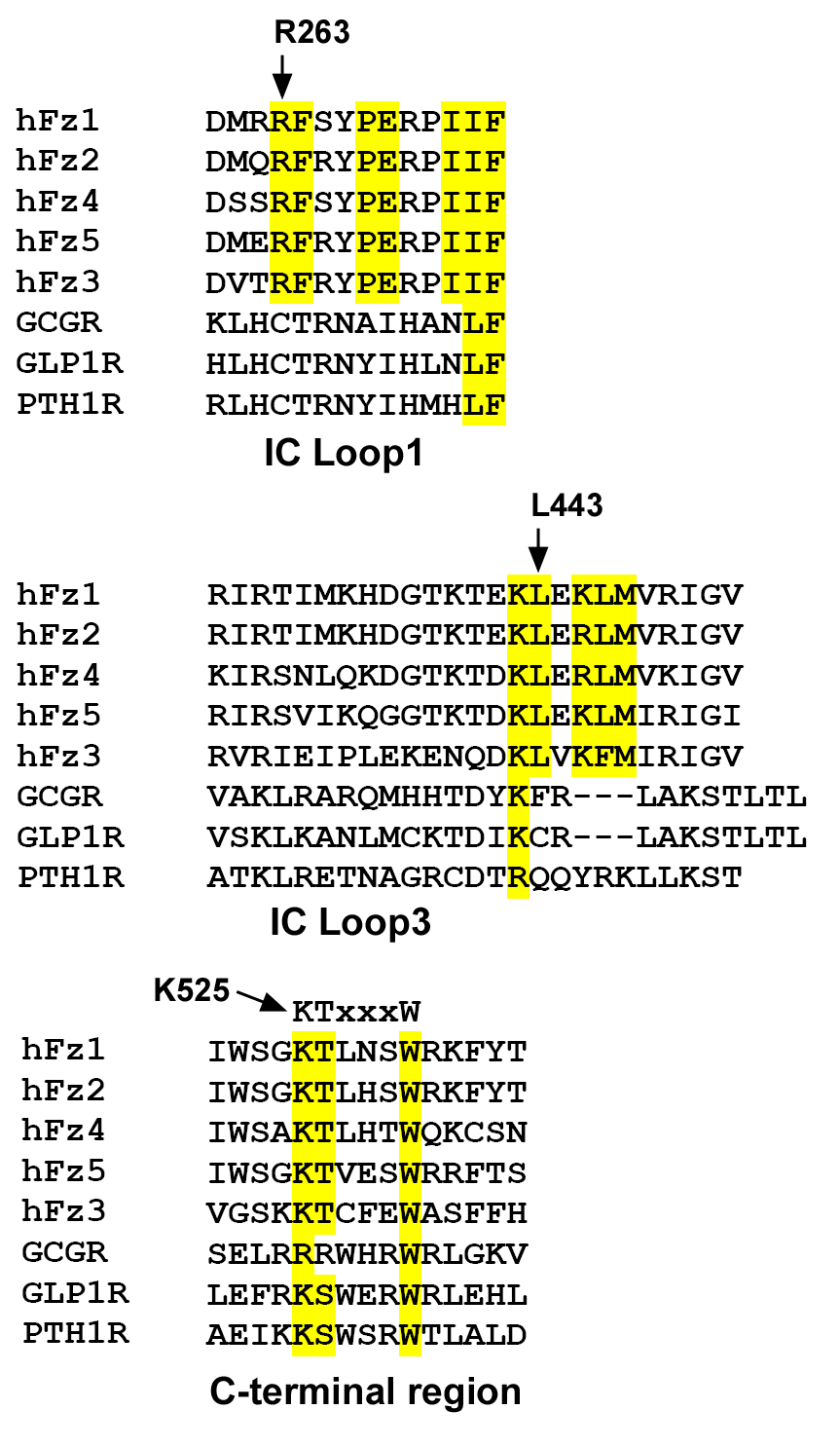

Supplement: Figure S3 — Sequence alignment of the intracellular loop 1 and 3, C-terminal region of Frizzled receptors and three class B GPCRs. The IC loops and C-terminal region were predicted by the HMMTOP server [35] and aligned by clustalW program [36]. The conserved residues critical for activation of Wnt/β-catenin signaling are highlighted in yellow based on previous studies [25]. Single mutations abolish Wnt/β-catenin signaling activity of human Frizzled 5 (Fz5) are indicated on the top of the alignment [25]. Residue number corresponds to human Fz5 sequence. (TIF) [file pone.0033676.s003.tif]

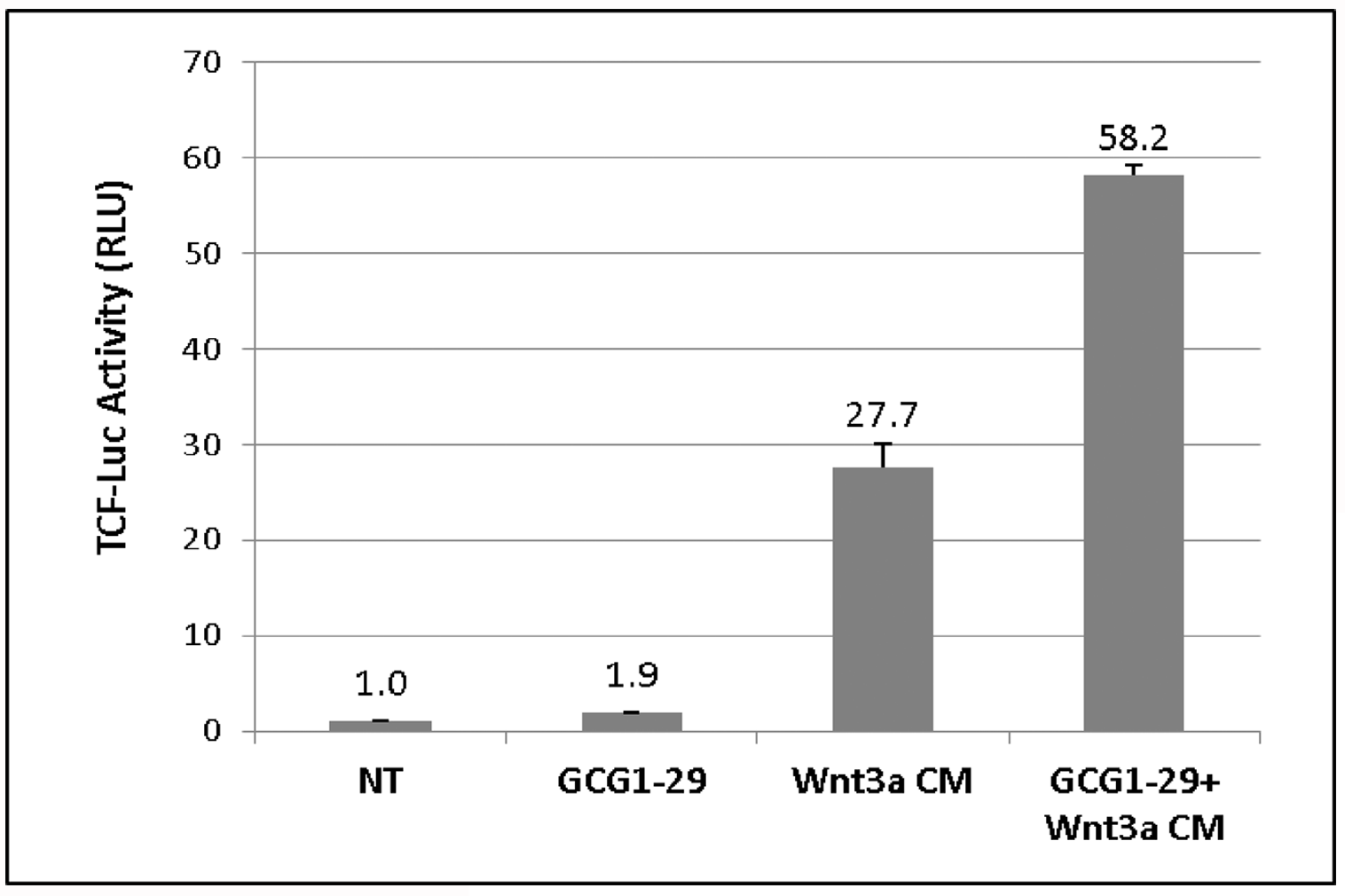

Supplement: Figure S4 — Effects of glucagon and Wnt3a on the TCF Luc reporter activity in the 293STF cells expressing GCGR and Lrp5. 293STF cells were transfected with 100 ng each of Lrp5 and GCGR plasmids and 10 ng of TKRlu (an internal control) on day 1. Cells were either left untreated, or treated with 50 nM GCG1-29, 10% Wnt3a Conditioned Media (CM) or 50 nM GCG1-29+10% Wnt3a CM on day 2. Cells were harvested on day 3 to measure the luciferase activity as described in the “materials and methods” section. The data label indicated the fold of induction compared with the non-treated group (NT). (TIF) [file pone.0033676.s004.tif]

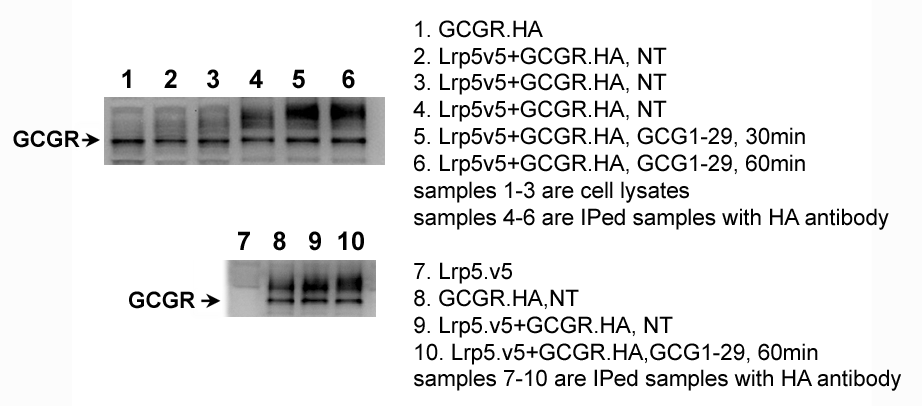

Supplement: Figure S5 — The immunoprecipitation experiment using HA antibody. HEK293 cells were transfected with 2000 ng of the indicated DNAs and treated with or without 50 nM GCG1-29 for the indicated time, similarly as in Fig. 7. Cells were harvested and lysed, and cell lysates with equal amounts of protein were used for western blot analysis for Lane 1–3. For lane 4–10, the cells were harvested and lysed, and equal amounts of lysate were immunoprecipitated with HA antibody. The antibody complex was pulled down by protein G beads. After pull-down, the beads were washed three times with 1× TBST and then incubated in 1× SDS sample buffer to release the bound proteins. The lysates were used for western blot analysis. (TIF) [file pone.0033676.s005.tif]
